# Supplementary material for: Valorization of the Red Algae Gelidium sesquipedale by Extracting a Broad Spectrum of Minor Compounds Using Green Approaches
Source: Mar Drugs. 2021 Oct 14;19(10):574. doi: 10.3390/md19100574 (PMC8539579; doi:10.3390/md19100574)
Supplement: Supplementary file 1 [file marinedrugs-19-00574-s001.zip › marinedrugs-1407826Zsl.pdf]

# Supplementary Materials: Valorization of the red algae *Gelidium sesquipedale* by extracting a broad spectrum of minor compounds using green approaches

**Natalia Castejón, Maroussia Parailloux, Aleksandra Izdebska, Ryszard Lobinski and Susana C. M. Fernandes**

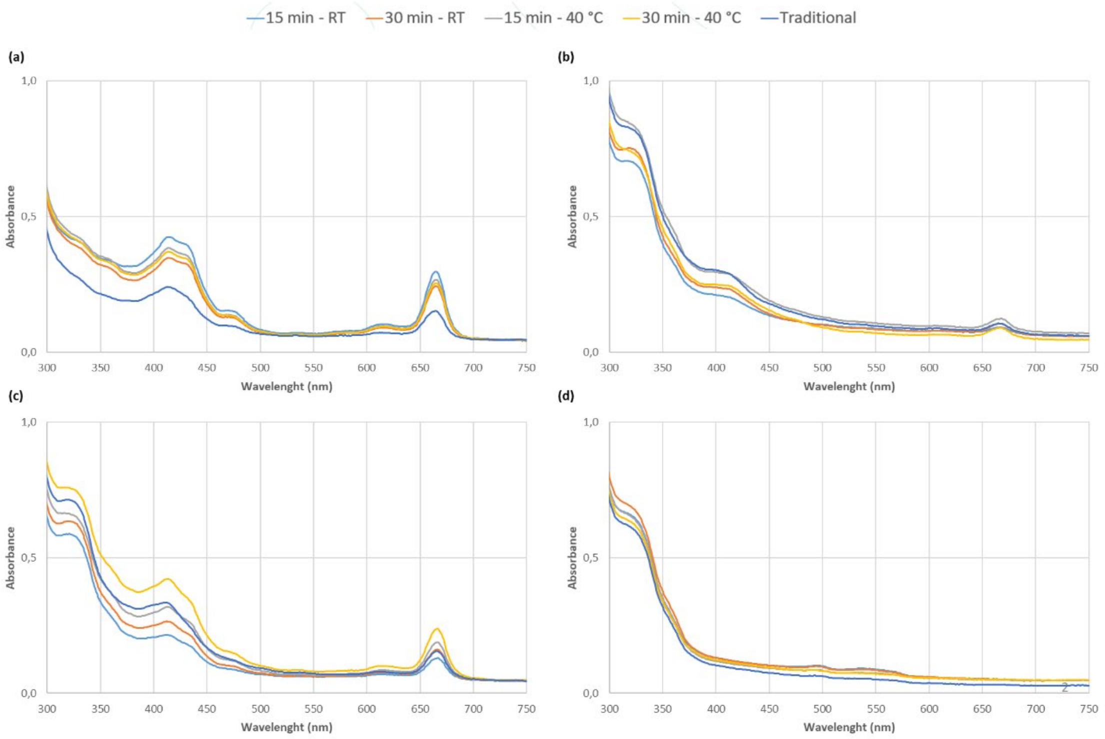


**Figure S1**. UV absorption spectra (λ=300 – 750 nm) of *Gelidium sesquipedale* extracts using ultrasound-assisted extraction and conventional solvent extraction techniques: ethanol extracts (a), ethanol:water (50:50 v/v) (b), ethanol:water (70:30 v/v) (c) and water extracts (d). The graphs show the mean values (n=3).

**Asterina-330**

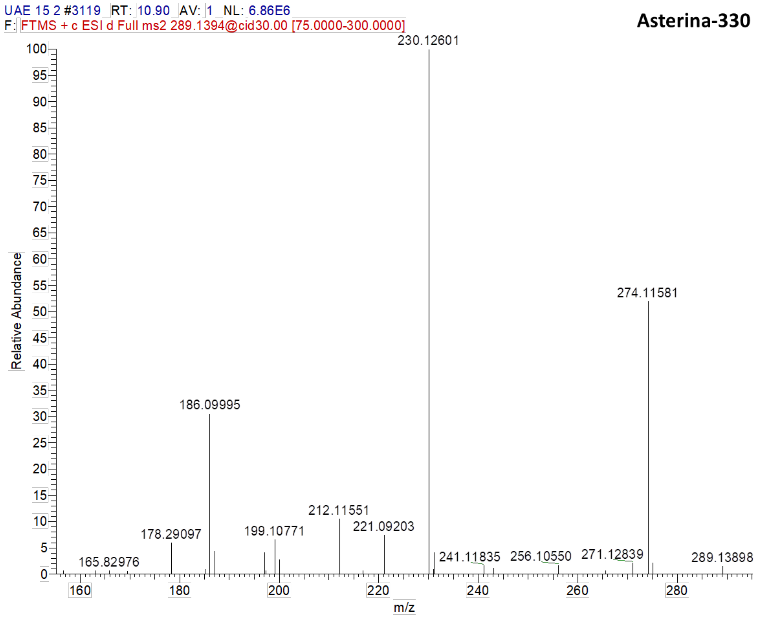


**In-Source fragmentation Asterina-330**

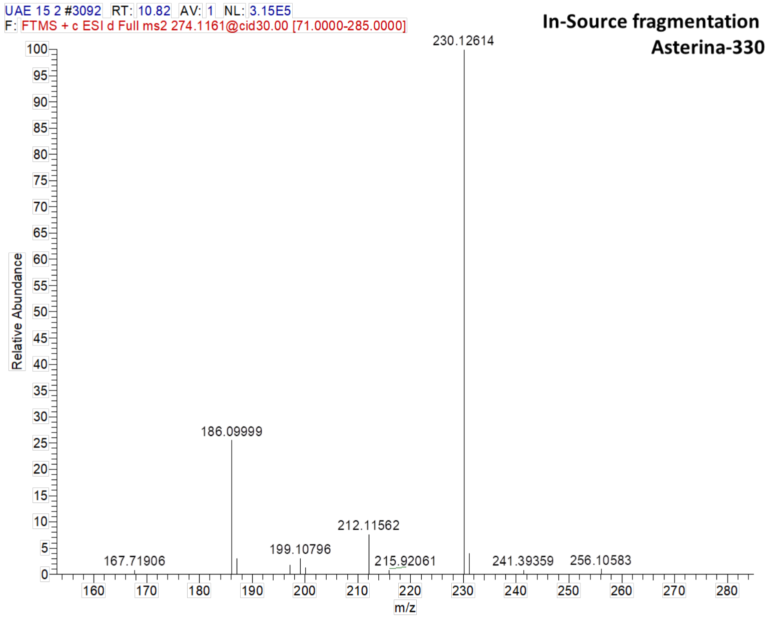


**Aplysiapalythine A**

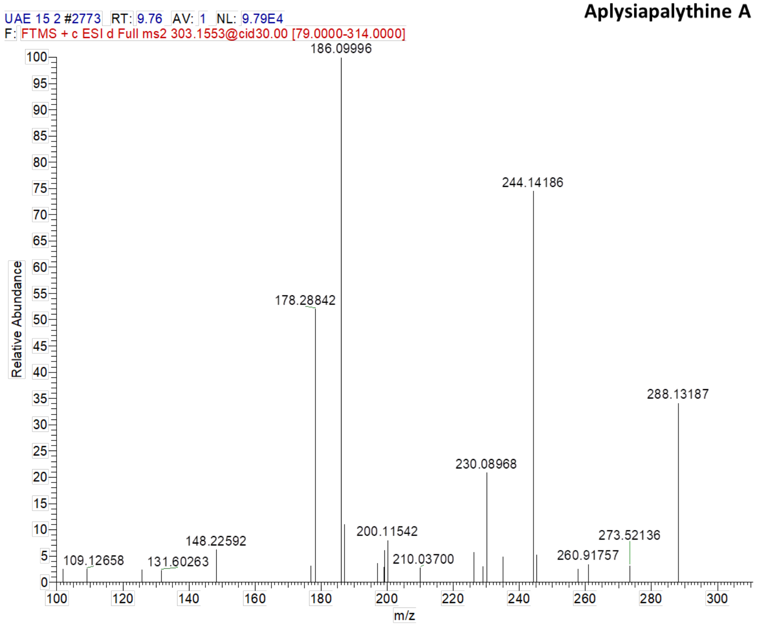


**Porpohyra-334**

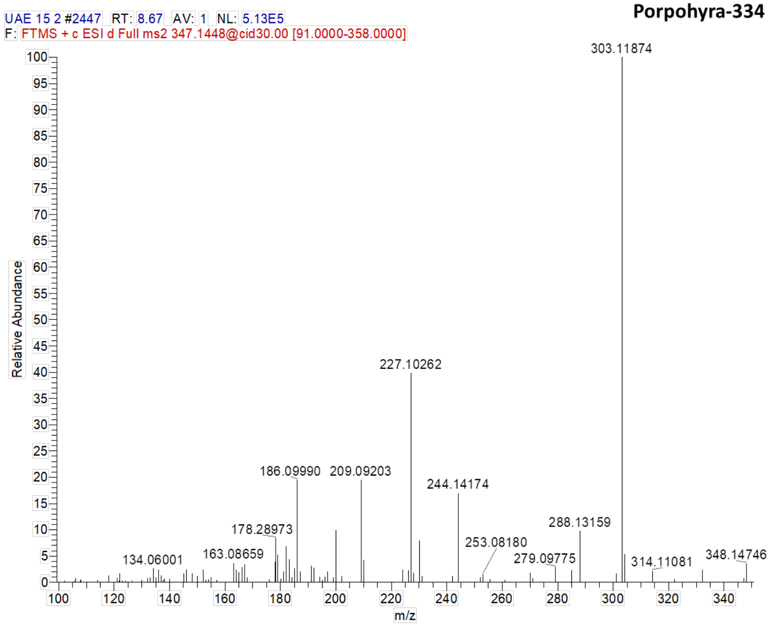

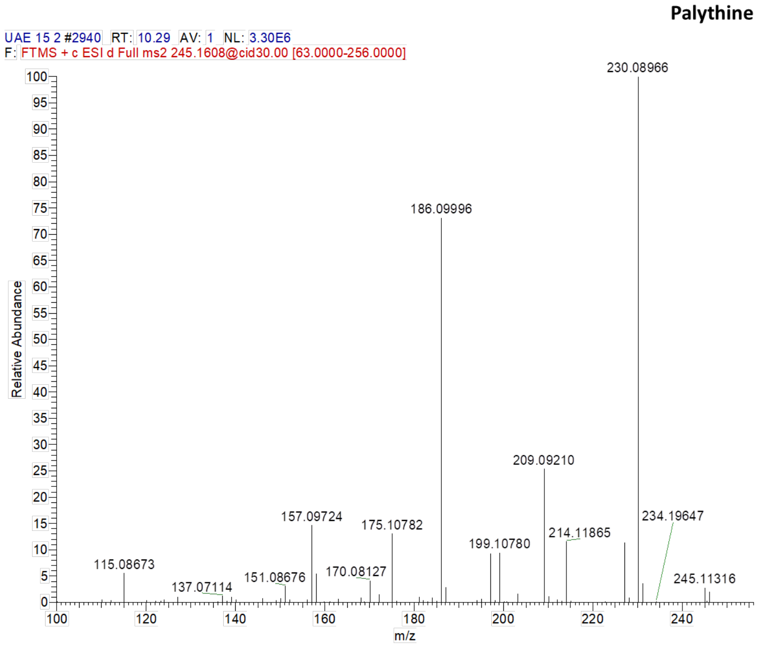
Palythine
UAE 15 2 #2940 RT: 10.29 AV: 1 NL: 3.30E6
F: FTMS + c ESI d Full ms2 245.1608@cid30.00 [63.0000-256.0000]
230.08966
186.09996
209.09210
157.09724
175.10782
199.10780
214.11865
234.19647
115.08673
137.07114
151.08676
170.08127
245.11316
Relative Abundance
m/z

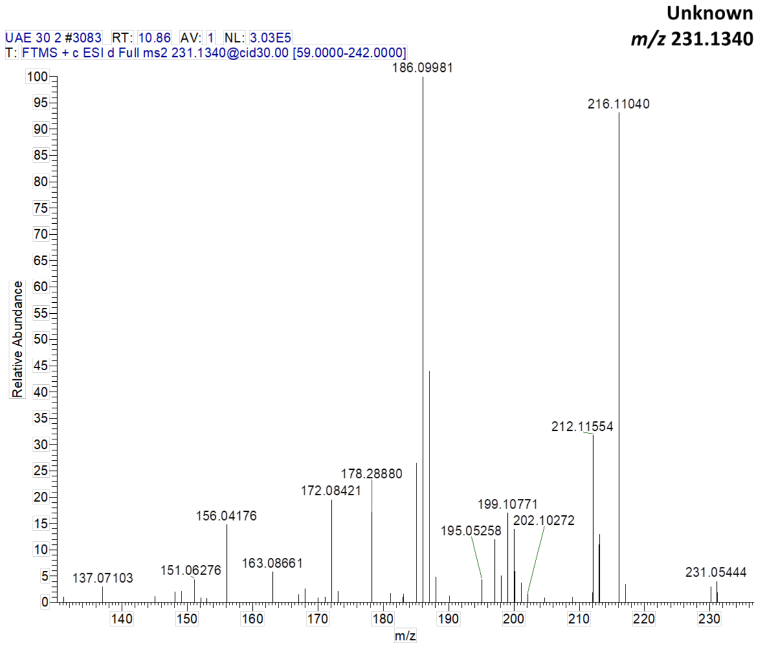
Unknown
m/z 231.1340
UAE 30 2 #3083 RT: 10.86 AV: 1 NL: 3.03E5
T: FTMS + c ESI d Full ms2 231.1340@cid30.00 [59.0000-242.0000]
186.09981
216.11040
212.11554
178.28880
172.08421
199.10771
156.04176
195.05258
202.10272
163.08661
151.06276
137.07103
231.05444
Relative Abundance
m/z

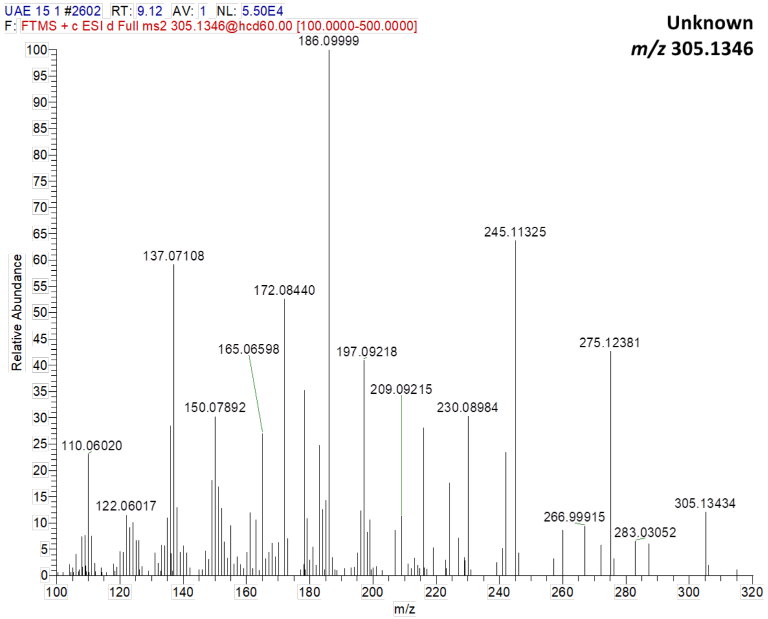
UAE 15 1 #2602 RT: 9.12 AV: 1 NL: 5.50E4
F: FTMS + c ESI d Full ms2 305.1346@hcd60.00 [100.0000-500.0000]
Unknown
m/z 305.1346
186.09999
245.11325
137.07108
172.08440
165.06598
197.09218
275.12381
209.09215
150.07892
230.08984
110.06020
122.06017
266.99915
283.03052
305.13434
Relative Abundance
m/z

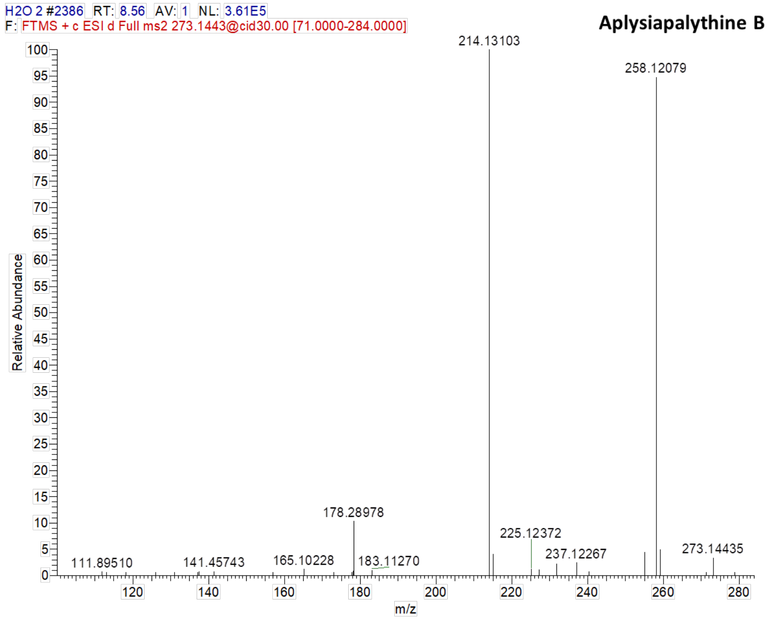
H2O 2 #2386 RT: 8.56 AV: 1 NL: 3.61E5
F: FTMS + c ESI d Full ms2 273.1443@cid30.00 [71.0000-284.0000]
Aplysiapalythine B
214.13103
258.12079
178.28978
225.12372
237.12267
273.14435
111.89510
141.45743
165.10228
183.11270
Relative Abundance
m/z

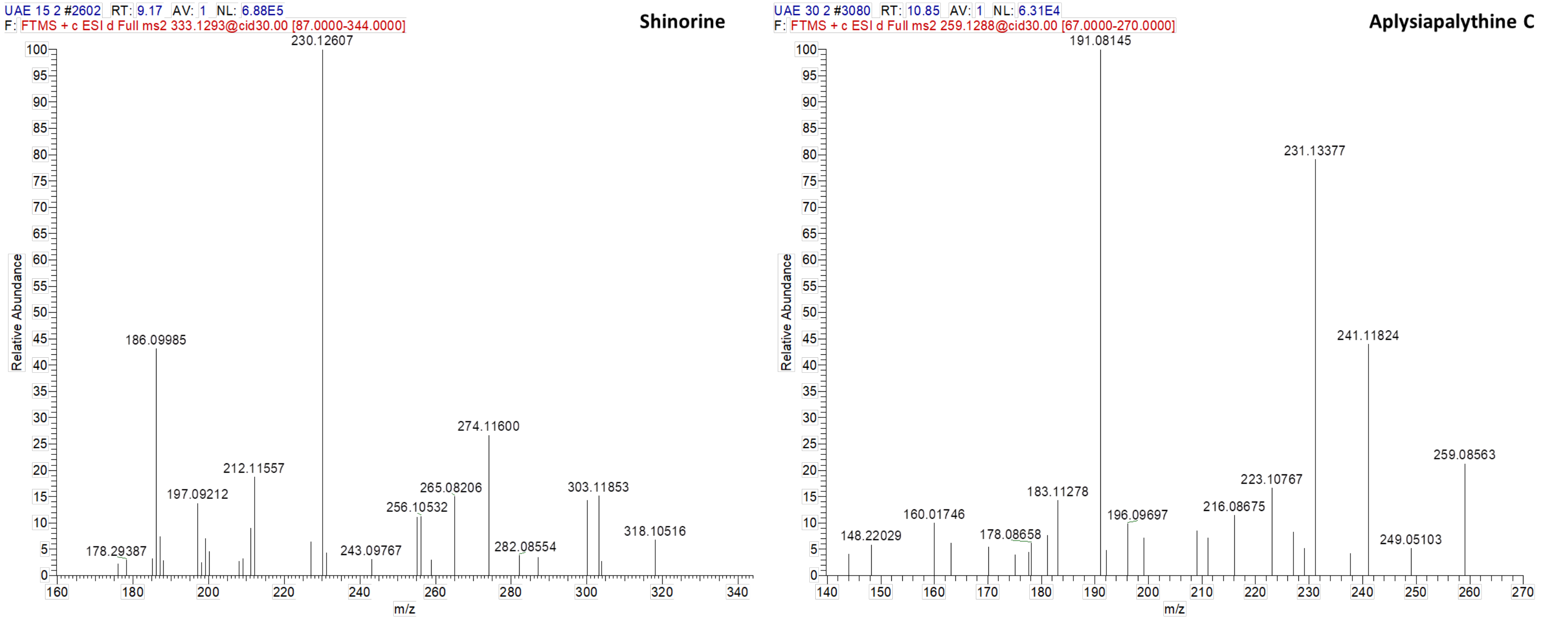


MS² Spectra of candidate-MAAs identified in the algal specie *Gelidium sesquipedale*. Here was shown fragmentation data collection acquired at CID30, HCD50 and HCD60+/-20.
